# Perfluorobutane Sulfonate (PFOS) Accumulation in Tissues of *Cherax quadricarinatus* and Its Toxicity Mechanism

**DOI:** 10.3390/toxics13040269

**Published:** 2025-04-01

**Authors:** Shuang Hong, Tian Zhu, Chengbin Liu, Yameng Li, Wei Song, Xianli Wang, Xiaoyu Liu, Hongzhuo Wang, Kepiao Li, Xiaolong Cao, Chunxia Yao, Weiwei Lv

**Affiliations:** 1 College of Fisheries and Life Science, Shanghai Ocean University, Shanghai 201306, China; 15802640428@163.com; 2 Institute for Agri-Food Standards and Testing Technology, Shanghai Academy of Agricultural Sciences, Shanghai 201403, China; liuchengbin@saas.sh.cn (C.L.); liyameng@saas.sh.cn (Y.L.); songwei890214@163.com (W.S.); wangxianli@saas.sh.cn (X.W.); 16678050954@163.com (X.L.); 15945539581@163.com (H.W.); 18985615583@163.com (K.L.); 18962293725@163.com (X.C.); 3 Centre for Marine and Coastal Studies, Universiti Sains Malaysia, Minden 11700, Penang, Malaysia; zt18913963586@163.com; 4 Eco-Environmental Protection Research Institute, Shanghai Academy of Agricultural Sciences, Shanghai 201403, China

**Keywords:** perfluoroctane sulfonate, *Cherax quadricarinatus*, transcriptomics, toxic effect

## Abstract

Perfluoroctane sulfonate (PFOS) is an emerging pollutant widely existing in aquatic environments that has attracted many scholars’ attention. *Cherax quadricarinatus* (*C. quadricarinatus*) are crustaceans that live in freshwater environments. This study aimed to investigate the long-term toxic exposure effect of PFOS on *C. quadricarinatus*. Three PFOS environment concentrations (1 ng/L, 100 ng/L, and 10 μg/L) were set for 28 days of exposure to *C. quadricarinatus*. The results indicated that PFOS was detected in the serum, muscle, and hepatopancreas of the *C. quadricarinatus*, and the order of accumulation levels was as follows: hepatopancreas > serum > muscle. Furthermore, transcriptomics showed that the function of differentially expressed genes (DEGs) in PFOS exposure groups was related to biological processes, metabolism, organic system, and immune response. The Kyoto Encyclopedia of Genes and Genomes enrichment analysis showed that DEGs were significantly enriched in the lysosome signaling pathway, retinol binding, fructose and mannose metabolism, and glutathione metabolism, etc., and the lysosome signaling pathway was the most significant, which indicated that lysosome signaling pathway is the key pathway for the toxic effects of PFOS on *C. quadricarinatus*.

## 1. Introduction

Poly- and perfluoroalkyl substances (PFASs) are a kind of synthetic organic fluoride that has been used in the industry and consumer products worldwide for more than 70 years [1]. Due to its stable chemical properties and long half-life, PFASs are difficult to decompose, thus called “forever chemicals” [2]. Among many PFAS, perfluorooctane sulfonate (PFOS) is difficult to remove and degrade in the environment due to its strong carbon–fluorine-bonded structure, causing long-term pollution [3]. Furthermore, PFOS is the main PFASs detected in both fresh and saltwater fish samples [4]. PFOS is more commonly found in the liver of aquatic organisms than other PFASs [5,6]. Environmental concentrations of PFOS have been reported several times in studies; the concentration of PFOS in the aquatic environment has been reported to be detected in the range of ng/L to μg/L [7]. In a previous study, PFOS concentrations ranging from 170 to 14,000 ng/L were detected in groundwater at a fire training area in Australia [8]. In addition, the presence of PFOS was detected even in human blood (0–1.6 μg/L) and breast milk (0.007–0.03 μg/L) [9,10]. Therefore, it is important to explore the toxicity of PFOS for ecological and human health.

Although the use of PFOS has been reduced and gradually replaced [3], its persistent environmental pollution remains a serious problem that threatens human and ecological health worldwide. For example, studies have shown that exposure to PFOS affects the expression of genes and proteins in the human cerebellum, causing neurotoxicity in the body. DNA damage was observed in carp after exposure to PFOS [11], induced cytotoxicity in zebrafish [12], and caused the disturbance of lipid metabolism in catfish [13]. Due to the wide distribution of PFOS in aquatic ecosystems, it is necessary to study its toxicity to aquatic organisms as the first group of organisms to which PFOS is exposed.

The red claw crayfish (*C. quadricarinatus*) is a kind of aquatic crustacean living in fresh water. It has delicate meat, a high edible ratio, low maintenance cost, and strong tolerance [14,15]. It is distributed in South China, the middle and lower reaches of the Yangtze River and some areas of Shandong Province. It is native to Oceania and is also distributed in the rivers of North Queensland, Australia. They show strong resistance to certain pollutants and environmental pressures, making them a good model for studying the long-term exposure effects of pollutants [16,17]. Currently, *C. quadricarinatus* has been widely cultivated and studied as a model organism [18]. By observing the survival, growth, and reproduction of *C. quadricarinatus* under different concentrations of pollutants, the long-term ecological impact of pollutants can be assessed. In addition, several studies have reported the response of *C. quadricarinatus* to exposure to toxic contaminants, including in studies on the toxic effects of other emerging contaminants and viruses, such as micro-/nanoplastics, microcystin, Vibrio parahaemolyticus, etc. [17,19,20]. However, as a typical pollutant widely existing in aquatic environments, PFOS is inevitably exposed to the growth environment of *C. quadricarinatus*. Compared to commonly used laboratory animals, *C. quadricarinatus* provides a new perspective to study toxicological mechanisms of action, which may reveal effects or pathways not observed in traditional models, thereby increasing our understanding of pollutant toxicity. Therefore, *C. quadricarinatus* was selected as the model organism, aimed to reveal the toxic effects of environmental PFOS on *C. quadricarinatus* and its molecular mechanism, and attempted to provide data support for the study of the toxic effects of PFOS on aquatic crustaceans.

Transcriptomics can accurately and directly reflect the changes in organisms at the gene level and is an important means to study cell phenotype and function [21]. Previous studies employing transcriptomic approaches have elucidated the toxicological effects of microplastics. For example, transcriptomic analyses have demonstrated that microplastics disrupt the expression of immune-related genes and inhibit antioxidant enzyme activity in *C. quadricarinatus*, ultimately inducing redox imbalance in vivo [16]. Transcriptomics was also used to assess the effects of polystyrene microspheres on oyster physiology [22]. Liver abnormalities are closely related to the occurrence of diseases and the toxicological effects of pollutants. Studying the physiological and biochemical changes in the liver is helpful in evaluating the toxic effects of pollutants. Therefore, many studies on PFASs focus on the physiological and biochemical changes in the liver to reveal its toxicological effects. Studies have shown that long-term exposure to PFOS can induce hepatic steatosis in zebrafish [23], long-term exposure to PFOS disrupts sex hormone levels and expression of related genes and impairs gonadal development [24]. Based on the above analysis, we mainly used transcriptomic differences combined with tissue slices to explore the exposure effect of PFOS on *C. quadricarinatus*.

By integrating transcriptomic profiling with environmentally relevant concentration gradients (1 ng/L, 100 ng/L, and 10 μg/L), this study systematically delineates the chronic molecular toxicity mechanisms of PFOS in non-model benthic crustaceans. The findings not only address the critical knowledge gap in the ecotoxicological data of PFOS at low exposure levels but also underscore the pivotal role of crustacean models in advancing research on emerging contaminants. Furthermore, this work provides a scientific foundation for developing informed risk mitigation strategies to safeguard aquatic ecosystems from PFOS-induced ecological hazards. This study can provide research data to examine the toxic effects of PFOS on aquatic crustaceans and help assess the environmental and ecological risks of PFOS.

## 2. Materials and Methods

### 2.1. Chemicals and Reagents

The reverse transcription and qPCR kits were purchased from Vazyme (Nanjing, China). Perfluorooctane sulfonate (CAS:2795-39-3, purity ≥ 98%) was purchased from Sigma-Aldrich (Darmstadt, Germany). During the experiment, the purchased potassium salt of perfluorooctane sulfonate was configured into a solution of 100 mg/L and added to the aerated water to prepare an exposed solution.

### 2.2. C. quadricarinatus Culture

The juvenile *C. quadricarinatus* was bought from Shanghai Yuanwang Aquatic Professional Cooperative and raised in the aquatic laboratory of the Shanghai Academy of Agricultural Sciences. Their initial body weight was 52.27 ± 1.45 g, and they were kept in freshwater laboratory conditions of 25 ± 1 °C, dissolved oxygen ≥ 7 mg/L, and PH 7.8–8.0. *C. quadricarinatus* were fed with commercial feed at 18:00 every night and fed 5% of their body weight. The exposed water was composed of tap water that had been aerated and dechlorinated for more than 24 h, and it was aerated continuously throughout the experiment. After a week of acclimation, the acclimated shrimps were randomly divided into 4 groups (0 ng/L (CK), 1 ng/L, 100 ng/L and 10 μg/L), and their exposure concentration and time were referred to the environmental concentration and previous studies [7]. Each group of *C. quadricarinatus* was raised in 3 polypropylene tanks, each tank (38 cm × 26.5 cm × 13 cm) filled with 10 L of water, and each box contained 4 juvenile female *C. quadricarinatus*. To maintain the concentration of PFOS, the exposure solution was completely changed every other day, and the water exposure test was conducted for 28 days. The whole culture experiment was carried out in a greenhouse.

### 2.3. Sample Collection

After the exposure experiment, the *C. quadricarinatus* were placed on ice and anesthetized with ice. The hepatopancreas of the *C. quadricarinatus* were completely removed with sterile tweezers and scissors and placed in a 2 mL frozen tube, frozen with liquid nitrogen, and stored in a −80 °C refrigerator for subsequent analysis. Three independent samples (N = 3) were randomly selected from each group for concentration detection, and six independent samples (N = 6) were randomly selected for transcriptomic analysis.

All animal experiments were conducted in strict compliance with internationally recognized ethical standards and the Guide for the Care and Use of Laboratory Animals (Ministry of Science and Technology of China, 2006, No. 398). The study protocol was reviewed and approved by the Independent Animal Ethics Committee of the Shanghai Academy of Agricultural Sciences (Approval No. SAASXM0925018).

### 2.4. Content Detection

The method of content detection is referred to that of [25]. Briefly, a precisely weighed 2.0 g (±0.1 g) sample aliquot was spiked with 20 μL of a 500 μg/L PFOS internal standard solution. Subsequently, 5 mL of 0.1% (*v*/*v*) aqueous formic acid (pH 2.3) and 15 mL of HPLC-grade acetonitrile were added sequentially. The mixture was then supplemented with 2.0 g of anhydrous sodium sulfate and 2.0 g of sodium chloride to induce phase separation. After thorough vortexing (3000 rpm, 30 s) and mechanical shaking (250 oscillations/min, 10 min), the mixture was centrifuged at 4000× *g* for 5 min at 20 °C.

A 10 mL aliquot of the organic supernatant was carefully transferred and concentrated to near-dryness (≤50 μL residual volume) under a controlled stream of nitrogen at 40 °C. The resulting residue was reconstituted in 1 mL of LC-MS grade methanol, vortexed for 30 s to ensure complete dissolution, and filtered through a 0.22 μm PVDF membrane filter prior to HPLC-MS/MS analysis. For the system blank (system blank analysis involves executing the complete experimental workflow—including mobile phase preparation, solvent equilibration, and sample pretreatment protocols—without introducing actual test specimens; this procedure serves as a critical quality control measure to identify and quantify systemic artifacts arising from instrumental carryover, reagent impurities, or ambient contaminants introduced during analytical operations), the same procedure was followed by adding 20 μL of the 500 μg/L PFOS internal standard, 5 mL of 0.1% formic acid in water, 15 mL of acetonitrile, 2 g of anhydrous sodium sulfate, and 2 g of sodium chloride. The mixture was vortexed, shaken, centrifuged, and processed identically, with the final solution also filtered through a 0.22 μm membrane filter for HPLC-MS/MS analysis.

### 2.5. Transcriptomics

Total RNA was isolated using the Trizol Reagent (Invitrogen Life Technologies, Carlsbad, CA, USA), after which the concentration, quality, and integrity were determined using a NanoDrop spectrophotometer ((Thermo Fisher Scientific, Waltham, MA, USA). Total RNA (3 μg) was used as the starting material for sequencing library construction. The workflow was performed as follows: polyadenylated mRNA was enriched from total RNA using poly-T oligo-attached magnetic beads (Illumina, Shanghai, China). Purified mRNA was fragmented into optimal sizes using divalent cations in Illumina proprietary fragmentation buffer at elevated temperature (94 °C, 5–7 min). First-strand cDNA synthesis was performed using random hexamer primers and SuperScript II Reverse Transcriptase (Thermo Fisher Scientific, Carlsbad, CA, USA), followed by second-strand synthesis using DNA Polymerase I and RNase H. The resulting double-stranded cDNA was end-repaired using T4 DNA polymerase and Klenow DNA polymerase to generate blunt ends, followed by 3′ end adenylation using Klenow exo- (3′→5′ exo minus).

Illumina-compatible PE adapter oligonucleotides were ligated to the adenylated fragments, and library fragments were size-selected (400–500 bp) using the AMPure XP system (Beckman Coulter, Brea, CA, USA). Adapter-ligated fragments were amplified by 15 cycles of PCR using Illumina PCR Primer Cocktail, followed by purification with the AMPure XP system. Library quality and quantity were assessed using the Agilent High Sensitivity DNA Kit on a Bioanalyzer 2100 system (Agilent Technologies, Santa Clara, CA, USA). Finally, the normalized libraries were sequenced on the NovaSeq 6000 platform (Illumina) using a 2 × 150 bp paired-end configuration.

#### 2.5.1. Data Quality Control

Following sequencing, raw image files were processed using the sequencing platform’s proprietary software (v0.22.0) to generate FASTQ-formatted raw data. Initial quality assessment revealed the presence of low-quality reads and adapter contamination, which could significantly compromise downstream analyses. To ensure data reliability, a rigorous quality filtering pipeline was implemented: (1) adapter sequences and low-quality bases at the 3′ end were trimmed using fastp (v0.22.0) with default parameters; (2) reads with an average Phred quality score below Q20 (indicating > 1% error probability) were systematically removed. These stringent quality control measures resulted in high-quality clean data, which served as the foundation for all subsequent bioinformatics analyses.

#### 2.5.2. Sequence Alignment to the Reference Genome

The reference genome and corresponding gene model annotation files were retrieved from the NCBI genome database. Genome indexing was performed using HISAT2 (v2.1.0) with default parameters, enabling efficient alignment of sequencing reads. Clean paired-end reads were then aligned to the indexed reference genome using HISAT2, which was selected as the alignment tool due to its superior performance in handling spliced alignments. Specifically, HISAT2′s ability to incorporate gene model annotations into its genome indexing process allows for more accurate alignment of reads spanning exon-exon junctions compared to non-splice-aware alignment tools. This approach ensures optimal mapping accuracy, particularly for eukaryotic transcriptomes where splice junction identification is critical for downstream analysis.

#### 2.5.3. Differential Expression Gene Analysis

Gene expression levels were quantified using HTSeq (v0.9.1) to calculate raw read counts for each gene. To enable cross-gene and cross-sample comparisons, expression levels were normalized using the Fragments Per Kilobase of transcript per Million mapped reads (FPKM) metric. For paired-end sequencing data, FPKM specifically quantifies the number of fragment pairs (where both reads map to the same transcript) per kilobase of gene length per million mapped fragments, providing a standardized measure of gene expression abundance.

Differential gene expression analysis was performed using DESeq2 (v1.38.3) with default parameters. Differentially expressed genes (DEGs) were identified based on the following stringent criteria: |log2FoldChange| > 1 (indicating at least a two-fold change in expression) and an adjusted *p*-value < 0.05 (Benjamini–Hochberg correction for multiple testing). This analytical pipeline was implemented following established methodologies described in previous studies [26], ensuring reproducibility and comparability of results.

#### 2.5.4. Enrichment Analysis

Top Gene Ontology (GO) (v2.50.0) was used for GO enrichment analysis, the *p* value was calculated by hypergeometric distribution method (the standard of significant enrichment was *p* < 0.05), and the GO term of significant enrichment of differential genes (all/up/down) was found to determine the main biological functions of differential genes. The Kyoto Encyclopedia of Genes and Genomes (KEGG) pathway enrichment analysis was performed using cluster Profiler (v4.6.0) software, focusing on significant enrichment pathways with *p* < 0.05. Gene Set Enrichment Analysis (GSEA) was performed using GSEA software (v4.1.0) to identify significantly enriched biological pathways and functional gene sets. Unlike traditional differential expression analysis, GSEA does not require predefined differential expression thresholds. Instead, all genes were ranked based on their expression patterns across the three sample groups, creating a continuous distribution of gene expression changes. The analysis then evaluated whether predefined gene sets showed statistically significant enrichment at the extremes (top or bottom) of this ranked list. This approach provides enhanced sensitivity for detecting subtle but coordinated changes in gene expression across biological pathways, particularly when individual gene-level changes may not reach strict significance thresholds. Statistical significance was determined using a permutation-based approach (1000 permutations) with a false discovery rate (FDR) < 0.25 considered significant, as recommended by the GSEA developers for maintaining an optimal balance between sensitivity and specificity.

### 2.6. qRT-PCR Validation Experiments

To test and verify the reliability of the transcriptome data, using quantitative PCR method with the results of the transcriptome 5 verify differentially expressed genes (Table 1. Nucleotide sequences and sources of primers used for qPCR.) in the analysis, using NCBI primer design online web site (https://www.ncbi.nlm.gov/tools/primer-blast (accessed on 9 October 2024)) for primer design. HiScript III RT SuperMix for qPCR (+gDNA wiper) kit was used for reverse transcription. The qPCR system was 20 μL. The 2 × ChamQ Universal SYBR qPCR Master Mix is 10 μL, primer1 and primer2 are 0.4 μL each, Template cDNA is 1 μL, and ddH_2_O is 1 μL. The reaction conditions were as follows: pre-denaturation at 95 °C for 30 s, denaturation at 95 °C for 10 s, denaturation at 60 °C for 30 s, 40 cycles, 3 replicates per sample, PCR amplification curve, and dissolution curve were confirmed after the reaction.

### 2.7. Data Analysis

The graphs were drawn using GraphPad Prism (v8.01), and significance analysis was performed using one-way analysis of variance (ANOVA). *p* < 0.05 is considered statistically significant, *p* < 0.01 as highly significant, *p* < 0.001 as extremely significant, and *p* < 0.0001 as ultra-highly significant. (* *p* < 0.05, ** *p* < 0.01, *** *p* < 0.001, **** *p* < 0.0001). In addition, linear regression analysis of Pearson coefficient was used to evaluate the correlation between RNA-seq and qRT-PCR methods on log2(FC) values.

## 3. Results and Discussion

### 3.1. The Content of PFOS in C. quadricarinatus

**Figure 1:**
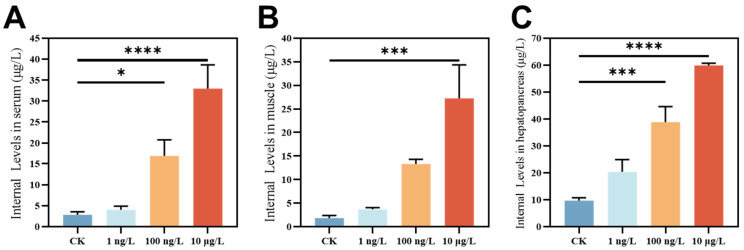
The levels of PFOS in different tissues of the *C. quadricarinatus* were determined, with the following distribution observed. (**A**) serum, (**B**) muscle, and (**C**) hepatopancreas. These values are expressed as mean ± SEM. Asterisks indicate statistical significance: * *p* < 0.05, *** *p* < 0.001, **** *p* < 0.0001; CK indicates 0 ng/L group.

We measured the accumulation levels of PFOS in the serum, muscle, and hepatopancreas tissues (Figure 1). As shown in Figure 1, we observed PFOS exposure in the CK (0 ng/L), which may be attributed to PFOS contamination accumulated during the wild growth of *Cherax quadricarinatus* (*C. quadricarinatus*). PFOS were not metabolized and thus persisted and bioaccumulated in their tissues. Furthermore, the figure demonstrates that PFOS accumulation intensified in *C. quadricarinatus* subjected to increasing levels. Furthermore, under all three exposure concentrations, the accumulation levels reached the microgram per liter (μg/L) range. Notably, PFOS was also detected at an environmentally relevant concentration of 1 ng/L; even at trace levels, the concentration level of PFOS in tissues is also shown. Studies have shown that long-chain PFASs, especially PFOS, are more likely to bioaccumulate in fish muscle tissue [27,28], and PFOS has also been detected in human serum [29]. Based on the observed bioaccumulation of PFOS and previous literature, we propose that this phenomenon is attributed to the role of the hepatopancreas in metabolism and detoxification. After entering the organism, PFOS is transported to the hepatopancreas for metabolic processing. However, due to its chemical stability, PFOS resists complete degradation, leading to its storage. Additionally, PFOS has a high affinity for binding to proteins [8,30], and the abundance of proteins in the hepatopancreas, serum, and muscle provides ample binding sites, further facilitating its store up. Our study demonstrates that PFOS can be stored up in organisms even at environmentally relevant concentrations, highlighting its potential impact on aquatic ecosystems.

### 3.2. Effects of PFOS Exposure to Hepatopancreas Gene Expression

**Figure 2:**
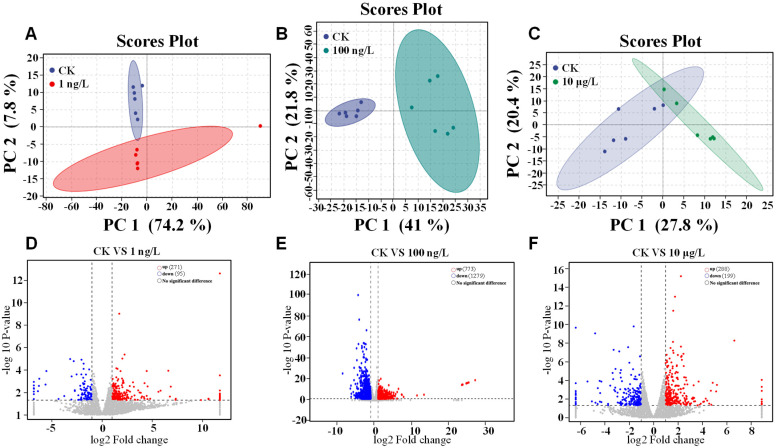
Transcriptomic analysis of *C. quadricarinatus* Hepatopancreatic. (**A**) PCA results of CK vs. 1 ng/L; (**B**) PCA results of CK (0 ng/L) vs. 100 ng/L; (**C**) PCA results of CK (0 ng/L) vs. 10 μg/L; (**D**) CK (0 ng/L) vs. 1 ng/L volcanic chart; (**E**) CK (0 ng/L) vs. 100 ng/L volcanic chart; (**F**) CK (0 ng/L) vs. 10 μg/L volcanic chart.

**Figure A1:**
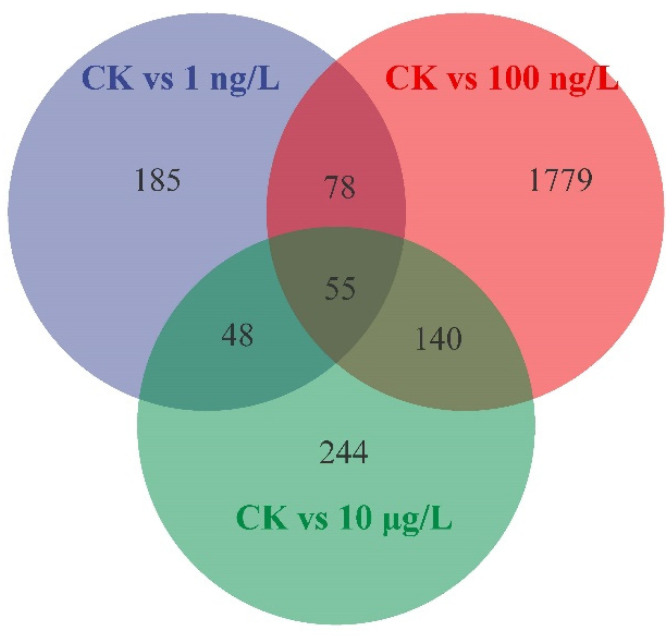
Venn diagram.

The results of PCA analysis (Figure 2A–C) preliminarily demonstrated the pollution effect of PFOS exposure on *C. quadricarinatus*. In Figure 2, we could see that CK (0 ng/L) has different principal components compared with both 1 ng/L 100 ng/L and 10 μg/L, which indicated that PFOS exposure had a certain impact on *C. quadricarinatus*. Furthermore, the Venn diagram (Figure A1) and volcano diagram (Figure 2D–F) also showed the same results as PCA. In the Venn diagram, we could see that there were certain significantly (*p* < 0.05) different genes among CK, 1 ng/L, 100 ng/L, and 10 μg/L. Volcano map also showed that the gene expression of CK and the exposed groups showed a certain opposite trend, and this trend was more obvious at a higher concentration of 100 ng/L. With *p* < 0.05 as the screening principle, compared with CK, a total of 366 DEGs were detected in the 1 ng/L group, including 271 up-regulated genes (74.04%) and 95 down-regulated genes (25.96%) (Figure 2D), a total of 271 DEGs were detected in the 100 ng/L group, including 773 up-regulated genes (37.67%) and 1279 down-regulated genes (62.33%), while 487 DEGs were detected in the 10 μg/L group. There were 288 up-regulated genes (59.14%) and 199 down-regulated genes (40.86%). These results showed that PFOS exposure caused a remarkable change in *C. quadricarinatus*, based on the difference in the number of DEGs, which suggested that the effect in 100 ng/L might be more obvious than in 1 ng/L and 10 μg/L. However, the manner by which PFOS produces its toxic effects still requires further analysis.

### 3.3. GO and KEGG Enrichment Analysis After PFOS Exposure

There are three aspects in the Gene Ontology (GO) enrichment analysis, which describe the molecular function of genes, the cellular component of cells, and the biological process involved. Based on all the pathways enriched in the GO enrichment analysis, we found that the three exposure groups were significantly enriched to 25, 47, and 61 pathways, respectively (*p* < 0.05) (Table A1). The results show that the changing pathways mainly focus on MF and BP, and CC only accounts for a very small number. However, the enrichment pathways of the three groups were mainly concentrated in the participating biological processes, and the number of enrichment pathways was 12, 19, and 31, respectively, indicating that the toxicological effects on *C. quadricarinatus* exposed to PFOS may focus on the biological processes involved. It has also been found in other studies that a high concentration of PFOS affects biological processes [31], and another study has also shown that these biological processes are also sensitive to exposure to pollutants in cod ovaries [32], which suggests that, after exposure to PFOS, we should pay attention to whether PFOS has negative effects on some pathways of biological pathways.

**Figure 3:**
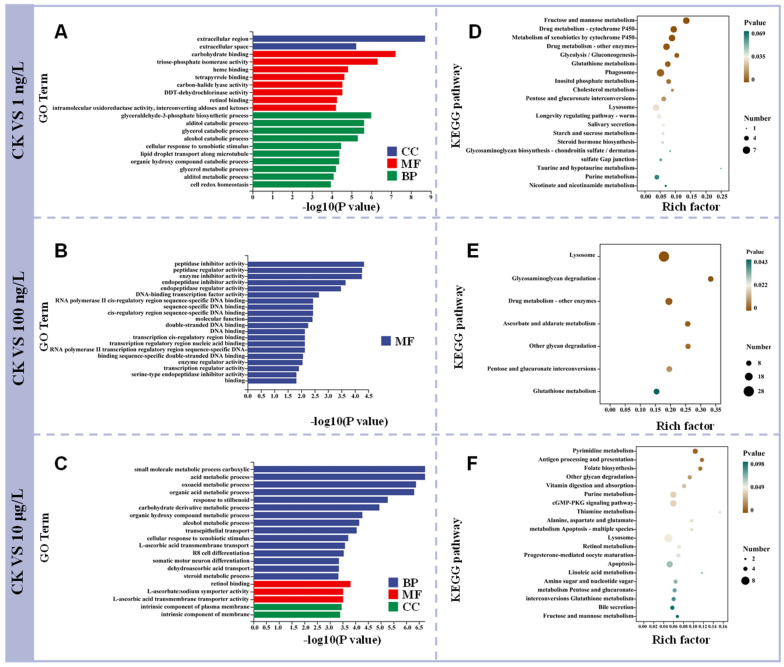
(**A**) Gene Ontology analysis of *C. quadricarinatus* hepatopancreas in CK (0 ng/L) vs. 1 ng/L; (**B**) Gene Ontology analysis of *C. quadricarinatus* hepatopancreas in CK (0 ng/L) vs. 100 ng/L; (**C**) Gene Ontology analysis of *C. quadricarinatus* hepatopancreas in CK (0 ng/L) vs. 10 μg/L; (**D**) KEGG enrichment analysis of *C. quadricarinatus* hepatopancreas in CK (0 ng/L) vs. 1 ng/L; (**E**) KEGG enrichment analysis of *C. quadricarinatus* hepatopancreas in CK (0 ng/L) vs. 10 μg/L. (**F**) KEGG enrichment analysis of *C. quadricarinatus* hepatopancreas in CK (0 ng/L) vs. 10 μg/L.

Figure 3 showed the enrichment results of the top 20 (*p* < 0.05) (Figure 3A,B). In addition, we could see that retinol binding pathway also appeared in both 1 ng/L and 10 μg/L, which indicated that these genes in retinol binding pathway could play key roles in biological processes involving retinol metabolism, transport, or function. There is evidence that retinoids are involved in the regulation of gene expression in fish by binding to nuclear receptors, thus controlling various aspects of growth, reproduction, and embryonic development [33]. In addition, it was found that PFOS interfered with the expression level of liver factors in mice, and retinol-binding protein-4 was significantly reduced [34], which also proves that PFOS may affect the liver metabolism of organisms through retinol binding. Additionally, in a comprehensive evaluation of the cumulative effects of anthropogenic and natural stressors on the health of yellow bass, retinol-binding proteins were selected as potential biomarkers [35]. However, there are few studies on the changes in retinol binding pathway caused by PFOS in organisms, which deserves our attention, and the retinol binding pathway may be a new target of PFOS action on *C. quadricarinatus*.

The result of KEGG enrichment analysis was showed in Figure 3C,D. We found that 104, 103 and 161 pathways were enriched in 1 ng/L 100 ng/L and 10 μg/L, respectively, and a total of 16 pathways were significantly enriched at 1 ng/L (*p* < 0.05), a total of 7 pathways were significantly enriched at 100 ng/L (*p* < 0.05), and a total of 11 pathways were significantly enriched at 10 μg/L (*p* < 0.05), and there were 4 pathways were observed to be co-enriched in both 1 ng/L and 10 μg/L, including fructose and mannose metabolism, glutathione metabolism, lysosome, and purine metabolism (*p* < 0.05), only lysosomal pathways were significantly enriched in the three exposure groups.

**Figure 4:**
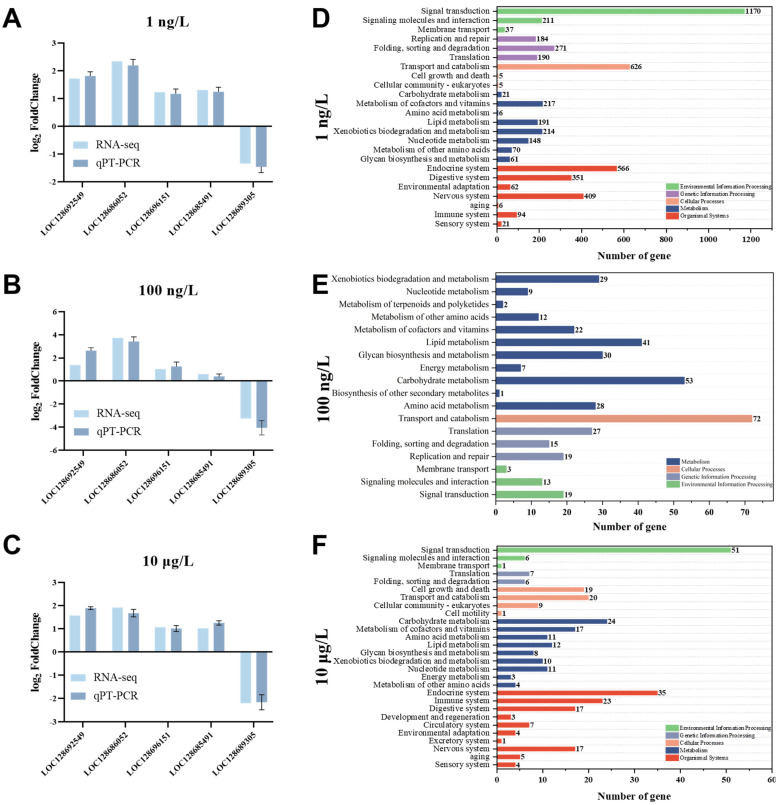
Transcriptomic enrichment of significant differential genes and pathways in *C. quadricarinatus* exposed to PFOS. (**A**–**C**) Expression levels of DEGs were revealed by qRT-PCR both in 1 ng/L, 100 ng/L and 10 μg/L. (**D**–**F**) KEGG pathway classification of different expression genes in 1 ng/L, 100 ng/L and 10 μg/L. The color indicates the primary classification in the KEGG enrichment pathway, and the ordinate represents the secondary classification in the KEGG functional enrichment pathway.

In addition, we analyzed the pathway categories and gene numbers by Kyoto Encyclopedia of Genes and Genomes (KEGG) functional enrichment (Figure 4C,D), and the results showed that the DEGs enriched in 1 ng/L were significantly (*p* < 0.05) more than other two exposure groups. In 1 ng/L, the number of DEGs in the environmental information processing pathway was higher, and the number of DEGs in the signal transduction pathway was as high as 1170. In addition, in both exposure groups, we found that pathways related to metabolism and organic systems were overwhelmingly present, which warrants our concern. In addition, by analyzing the secondary classification of KEGG pathways, we found that the 10 μg/L still had KEGG pathway types that were not enriched in the 1 ng/L, such as the excretory system, circulatory system, excretory system, development and regeneration, and cell motility, which indicated that PFOS would have a greater impact on the body and affect the normal physiological functions of organisms when exposed to 10 μg/L PFOS. Notably, the activation and adaptation of emergency responses are associated with alterations in signaling pathways, or gene expression, which represent the earliest response of organisms to exposure to pollutants, reflecting the mode of action of exogenous organisms [36,37]. In this study, PFOS exposure caused disturbances in the signaling pathway of *C. quadricarinatus* to varying degrees; however, this phenomenon has also been seen in studies of exposure to other pollutants. For example, previous studies have found that in the case of Vibrio parahaemolyticus infection, the immune response of *C. quadricarinatus* was induced, and the expression of immune-related genes was changed [38]. Microplastics, another emerging pollutant, were also found to have a certain impact on the hepatopancreas of *C. quadricarinatus*, including inhibiting the growth of *C. quadricarinatus* and causing changes in lipid metabolism [16]. Some studies have also shown that nanoplastics affect the expression of immune-related genes and the activity of antioxidant enzymes in *C. quadricarinatus*, thus changing the composition and diversity of intestinal microbiota [17]. At the transcriptomic level, microcystin caused significant changes in genes related to detoxification, immunity, and apoptosis [19]. In addition, the toxic effects caused by PFOS exposure are also well known. Acute exposure to PFOS revealed significant gene expression changes in carbohydrate metabolism, energy metabolism, amino acid metabolism, lipid metabolism, and protein biosynthesis in Manila clams [39]. PFOS exposure caused significant changes in miRNA expression patterns of zebrafish embryo species involved in development, apoptosis, cell signaling pathways, cell cycle progression, and proliferation [40]. These observations, together with the results of this study, suggest that exposure to PFOS at environmentally relevant concentrations may impair the normal physiological function of *C. quadricarinatus*.

### 3.4. Validation of Transcriptomic Results

We performed RT-qPCR analysis of key DEGs co-enriched by the three exposed groups in the transcriptome to verify the transcriptome results, as shown in Figure 4A–C. The results showed that four genes were up-regulated and one gene was down-regulated, which was consistent with the transcriptomic analysis results. These results also indicated that the transcriptomic results were reasonable and reliable.

### 3.5. Changes in Lysosome Signaling Pathway

**Figure 5:**
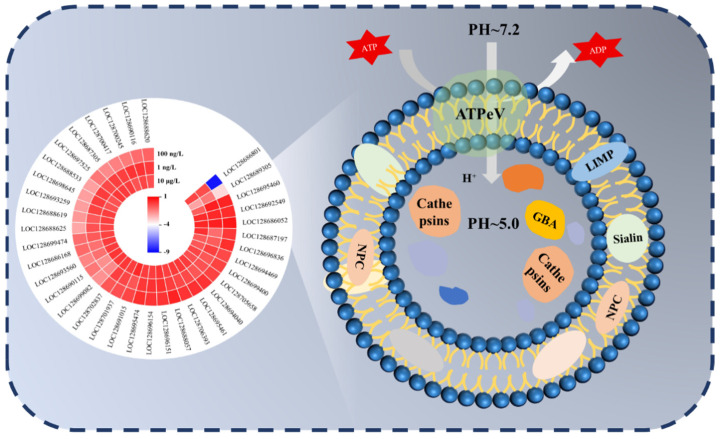
DEG expression in lysosomal signaling pathway.

Lysosomes are small intracellular vesicles containing a variety of hydrolytic enzymes, which are found in almost all types of eukaryotic cells, and they play a crucial role in the degradation, recycling, autophagy, and disposal of foreign substances in the cell [41,42]. We analyzed that PFOS exposure caused hepatopancreas cell apoptosis, which led to a significant expression of lysosomes. The results indicated that PFOS exposed to water had toxic effects on the hepatopancreas of *C. quadricarinatus*. As can be seen from Figure 5, PFOS exposure caused changes in many genes in the lysosomal pathway. Meanwhile, PFOS exposure mainly affected the expression of lysosomal acid hydrolases and lysosomal membrane proteins. Significant differences in cathepsins and membrane proteins (NPC) between the three exposure groups and the CK group suggest that these three substances may be important targets for PFOS to act on lysosomes and should attract our attention. In addition to these two substances, GBA, Sialin, and LIMP were significantly different in the 10 μg/L group, which may be due to the more intense immune response caused by a high concentration of PFOS, resulting in significant changes in the lysosome. In other studies, we also found that other pollutants caused lysosome damage on *C. quadricarinatus*; for example, Aeromonas veronii caused significant lysosome enrichment in the case of A. veronii infection, causing the body to produce an immune response [18]. Other studies have also found that PFOS induces mitochondrial dysfunction by blocking autophagic lysosomal degradation, leading to the myocardial cytotoxicity of embryonic stem cells [43]. By acridine orange staining, ref. [44] found that induced lysosomal membrane permeability (LMP). PFOS increased membrane stability and lysosome activity in tetrahymena [45]. PFOS induces apoptosis signaling and proteolysis in human lymphocytes through reactive oxygen species (ROS) and mediates mitochondrial dysfunction and lysosomal membrane labialization [46]. Studies observed a gradual increase in lysosomal iron in L-O2 cells after exposure to PFOS 0.5–24 h [47]. There are various indications that we must pay attention to the harm caused by PFOS exposure to aquatic animals. In conclusion, evidence of significant changes in hepatopancreas cell apoptosis and functional indicators reinforces the notion that chronic exposure to PFOS induces hepatopancreas toxicity in *C. quadricarinatus*.

## 4. Conclusions

This study integrated transcriptomics to study the toxicological effects and mechanisms of long-term exposure of *C. quadricarinatus* to PFOS in water at the molecular level. Transcriptomics analysis identified the specific genes and pathways affected by PFOS under PFOS exposure, and the results of Gene Ontology (GO) and Kyoto Encyclopedia of Genes and Genomes (KEGG) enrichment analysis revealed the physiological functions of PFOS in the hepatopancreas of *C. quadricarinatus* and the toxicological effects of related pathways; the lysosome is a key pathway for the toxic effects of PFOS exposure on *C. quadricarinatus* hepatopancreas. The results of this study revealed the effects of three environmentally relevant concentrations of PFOS on aquatic crustaceans, which can provide a reference for further investigation into the toxic effects and mechanisms of environmental PFOS on aquatic organisms.

## Figures and Tables

**Table 1:** Nucleotide sequences and sources of primers used for qPCR.

Gene	Forward Sequence (5′-3′)	Reserve Sequence (5′-3′)
LOC128692549	CCCACACCGTCTACCAAGTC	GGAGTGCCCATAGTCTCGC
LOC128686052	ACGGAGACATGCTCCATCA	GTGGCTCCACTACCACAGTC
LOC128696151	GGTACGAGTCTCAGTGCGTC	CAGGAGACTCCACTGCCTTG
LOC128685491	TGGGGATCACTCCATCACCT	GGTGACGATTTTGACTCAGCA
LOC128689305	GCTTACAGCTTCTGGGGTGT	TGCTTGCCCGAGATTAGACG
β-actin	AAATCCTACGAGCTTCCTGACG	TACCACAAGATTCCATGCCCAA

## Data Availability

The original contributions presented in the study are included in the article and Appendix A and Appendix B. Further inquiries can be directed to the corresponding author.

## Abbreviations

The following abbreviations are used in this manuscript:

PFOS	Perfluorobutane sulfonate
*C. quadricarinatus*	*Cherax quadricarinatus*
DEGs	Differentially expressed genes
PFASs	Poly- and perfluoroalkyl substances
GO	Gene Ontology
KEGG	Kyoto Encyclopedia of Genes and Genomes
qRT-PCR	Quantitative real-time fluorescent quantitative PCR
ANOVA	One-way analysis of variance

## Appendix A

**Table A1:** Summary of the number of classified pathways in GO enrichment analysis.

Group	1 ng/L	100 ng/L
MF	11	27
CC	2	1
BP	12	19
total	25	47
